# We need more research on inpatient group therapy: A call to action

**DOI:** 10.1017/S0033291725000960

**Published:** 2025-05-16

**Authors:** Mariah T. Hawes, Stephanie Marcello, Evan M. Kleiman

**Affiliations:** 1 Rutgers University Behavioral Health Care, USA; 2 Department of Psychology, Rutgers, The State University of New Jersey

**Keywords:** inpatient group therapy, inpatient psychotherapy, psychiatric hospitalization, psychiatric inpatient treatment

On a given day, over 180,000 people in the U.S. are hospitalized in a psychiatric inpatient or residential care unit (Lutterman, 2022). People enter psychiatric inpatient treatment, voluntarily or involuntarily, in periods of crisis, when their safety, the safety of others, or their ability to care for themselves is significantly impaired by mental health problems like active psychosis, mania, substance use, or suicidal thoughts and behaviors. A primary aim of inpatient treatment is rapid stabilization and movement to lower levels of care (e.g., partial hospitalization, outpatient treatment; Glick, Sharfstein, & Schwartz, 2011; Sharfstein, 2009); however, failed care transitions are common, with an estimated 42–51% of patients not attending any mental health visits within 30 days of discharge (Olfson, Marcus, & Doshi, 2010; Smith et al., 2017; Stein et al., 2007). Psychiatric hospitalization is clearly a critical time to intervene with acute mental health problems and may be the first and only treatment that some individuals receive (Salinsky & Loftis, 2007). Yet, it is the level of care in which patients are least likely to encounter evidence-based *psychological* treatment (EBT).

There are a few key factors contributing to the lack of psychological EBT in inpatient treatment: (1) the average length of psychiatric hospital stay in the U.S. (~3–7 days) has decreased substantially over the past 50 years and is now much shorter than most EBT protocols; (2) inpatient psychological treatment consists almost exclusively of group therapy; (3) however, the inpatient staff conducting group therapy are less likely to have training in psychological EBTs than the clinicians traditionally employed in clinical trials (i.e., psychologists); which culminates in (4) very little research has been conducted on inpatient groups designed for the current era of ultra-brief inpatient stays. In this article, we outline these factors in greater detail to underscore the dire need for more research on inpatient psychological EBT. Rather than arguing for an increase in the length of stay or a change in format (individual versus group) of psychological services offered in inpatient care, which would not be realistic given financial and logistical constraints, we argue that there is a need to adapt existing EBTs for inpatient group therapy and research studying the effectiveness and implementation of such interventions. Whether inpatient care is the most effective approach to treating acute mental health problems is a separate discussion covered elsewhere (e.g., Ward-Ciesielski & Rizvi, 2021). The reality is that many individuals require psychiatric hospitalization at some point in their lives, during which they will spend a substantial amount of time in group therapy. These groups are a missed opportunity to provide evidence-based care, which will certainly lead to improved inpatient care.

## The length of inpatient stay has substantially decreased in the U.S.

Psychiatric inpatient treatment has declined drastically in the U.S. over the past several decades, starting with the deinstitutionalization movement of the 1960s and 1970s. This movement was motivated by a combination of factors, including concern over inhumane conditions in long-term care facilities, a shifting philosophy towards less-restrictive mental health treatment, and the emergence of effective psychotropic medications that enabled care for many individuals with severe mental illness to be managed in community settings (e.g., outpatient clinics, partial hospitalization programs). Several key policy changes over the past 50 years have redirected federal funding from hospitals to community-based care, starting with President Kennedy’s 1963 Community Mental Health Act and the enactment of Medicaid in 1965 (Lutterman, 2022; Salinsky & Loftis, 2007). Similar deinstitutionalize movements were happening internationally (Fakhoury & Priebe, 2002) with the enactment of similar policies, like the Mental Health Act of 1959 in the U.K., which removed the distinction between psychiatric and other hospitals (Turner, 2004), the 1978 Basglia Law in Italy mandating the closure of all psychiatric hospitals (Badano, 2024), the Psychiatric Reform Law of 2001 in Brazil that expanded community-based services (Taborda, 2013), and the Mental Health Care Act of 2002 in South Africa emphasizing treatment in the least restrictive environment (Szabo & Kaliski, 2017). Since 1970, the number of individuals in inpatient and residential care beds on a given day in the U.S. has reduced by over 60%, from 471,451 to 187,877 in 2018, which was largely driven by decreases in state and county psychiatric hospital beds (−90.3%), while private psychiatric hospital capacity increased (+396.2%). Despite increases in some settings, accounting for population growth, the overall rate of patients in inpatient care declined by 76% from 236.8 to 56.8 per 100,000 between 1970 and 2018 (Lutterman, 2022).

Reductions in capacity and increased emphasis on less restrictive community-based treatment led to a substantial decrease in the duration of hospitalization (Lave, 2003). The average length of hospital stay shortened drastically from long-term care lasting years/decades prior to the 1950s to hospital stays lasting weeks/months in the latter half of the 20th century. The rise in managed care starting in the 1980s applied further pressure to shorten the length of hospital stays to reduce costs and increase patient turnover (Geller, Fisher, McDermeit, & Brown, 1998; Lave, 2003). Although variable depending on the setting, region, and patient characteristics, estimates for the current average length of inpatient stays range from 3 to 7 days (Adepoju, Kim, & Starks, 2022; Shah, Leontieva, & Megna, 2020), which is a reduction even relative to the early 2000s (~10 days; Lave, 2003; Tulloch, Fearon, & David, 2011). The current inpatient care model in the U.S. is a new age of ultra-brief hospital stays focused on crisis stabilization and rapid movement to lower levels of care (Glick, Sharfstein, & Schwartz, 2011; Sharfstein, 2009). This shift has forced providers to adapt psychological treatments to fit within a timeline they are not designed for without relevant research to inform these changes (Snyder, Clark, & Jones, 2012).

## Group therapy is the predominant mode of psychological treatment in inpatient care

Inpatient hospitalization is the most expensive form of mental health treatment, and the majority of these costs are attributable to staffing (Salinsky & Loftis, 2007). Different from other acute medical care settings where expensive equipment, materials, or space (e.g., imaging machines, laboratory testing, operating room suites) may make up a substantial portion of costs, staff time accounts for over 85% of inpatient psychiatric treatment costs (Cromwell et al., 2004). This time involves activities such as assessment, medication management, psychotherapy, and behavioral monitoring to ensure safety. In outpatient treatment, these activities are paid on a per-service basis (e.g., per therapy hour). However, as a result of the Medicare, Medicaid, and SCHIP (Supplemental Children’s Health Insurance Program) Balanced Refinement Act of 1999’s mandate to implement a per-diem reimbursement rate for inpatient psychiatric stays (Balanced Budget Refinement Act, 1999), inpatient treatment is now generally covered at a per-diem rate across providers, which incentivizes prioritization of services that are efficient with respect to staff time. Group therapy is a particularly efficient service because multiple patients can receive treatment from a single provider simultaneously.

The per-diem payment system, coupled with policy mandates pressuring state hospitals to increase treatment offered during inpatient stays, has led to the current norm in which group therapy is the main modality of psychological treatment in inpatient units, and access to individual therapy is limited (Cook, Arechiga, Dobson, & Boyd, 2014; Scaturo, 2004). For example, in 2003, state hospitals in North Carolina were ordered to increase the minimum hours of active treatments provided to all patients to 20 hours/week and responded by ramping up group programming so that patients were spending several hours/day in therapy groups (Snyder, Clark, & Jones, 2012). Although there have been recent efforts to develop ultra-brief psychological EBTs for inpatient care, these treatments have almost exclusively been individual therapy models (e.g., Diefenbach et al., 2024; Paterson et al., 2018; Tyrberg & Klintwall, 2022). As we detail below, research on inpatient psychological treatment in general is very limited, but for inpatient groups, this is even more so the case.

## Inpatient staff delivering psychological treatment have limited training in EBT

Another consequence of the cost-conscious managed care era of U.S. healthcare is that hospitals are incentivized to hire staff with the lowest credentials (and, thus, who can be paid the lowest rate) who can provide “equivalent” services. Lack of research leads to ambiguity about the level of training necessary for treatment to be implemented in a way that it can reach a standard of evidence-based care. Consequently, inpatient units employ few psychologists, primarily relying on masters-level clinicians (and in some cases, bachelor-level clinicians) to provide psychological services, who are likely not trained to deliver the type of EBTs that currently exist (Berry et al., 2022; Dandan, Mansour, & Diab, 2024; Pudalov, Swogger, & Wittink, 2018). A recent systematic review of staff perspectives on the barriers and facilitators to the implementation of psychological treatments in inpatient settings identified a lack of staff training in psychological EBTs as one of the main barriers (Evlat, Wood, & Glover, 2021). This is especially the case for group therapy. Irvin Yalom, who is widely considered an authority on group therapy and has written one of the only clinical texts on inpatient group therapy, cautions that lack of hospital leadership buy-in to the value of group therapy leads to a number of consequences that undermine the effectiveness of groups (e.g., inconsistent scheduling, frequently pulling members out of the group to complete administrative tasks), including assigning groups to be led by staff with the least experience with psychological treatments (e.g., nurses; Yalom, 1983; Yalom & Leszcz, 2020).

Across settings, clinical trials of psychological treatments overwhelmingly rely on psychologists and doctoral trainees to administer treatments. Research on inpatient group therapy is no exception, and the vast majority of inpatient therapy research has involved group interventions implemented by Ph.D.-level psychologists (e.g., Boynton & Sanderson, 2022; Esposito-Smythers, McClung, & Fairlie, 2006; Heriot-Maitland, Vidal, Ball, & Irons, 2014; Raune & Daddi, 2011). The norm of masters- or bachelors-level clinicians and other staff (e.g., nurses) providing group therapy on inpatient units is unlikely to change without major shifts in financial incentives or regulations on which disciplines can provide group therapy. We are not recommending such regulations be implemented; rather, we argue that clinical trials of inpatient group therapy should prioritize utilizing staff who are typically responsible for running groups in the real world. Given the often lack of background in psychological EBTs of non-psychologist staff, training may be a necessary prerequisite. Research on training in psychological EBTs is itself very limited (Frank, Becker-Haimes, & Kendall, 2020). Training multidisciplinary staff to deliver psychological EBTs is another area of research in dire need of development and is virtually nonexistent for the implementation of inpatient group therapy.

## Research on ultra-brief inpatient group therapy is extremely limited

Research on inpatient group therapy had a period of proliferation in the 1970s/80s, following a trend of increased interest in group therapy more broadly. This research has been summarized in meta-analyses (Burlingame, Fuhriman, & Mosier, 2003; Kösters, Burlingame, Nachtigall, & Strauss, 2006), which supported the effectiveness of inpatient group therapy, though effect sizes were notably smaller compared to outpatient groups (Burlingame, Fuhriman, & Mosier, 2003). Even though they were published 20 years ago, both meta-analyses noted an already significant decline in inpatient group research in the U.S. since the 1980s, while research increased in other countries. For example, 60% of the studies included in Kösters, Burlingame, Nachtigall, and Strauss’s (2006) meta-analysis were published in Germany, where the average length of stay is >5 weeks (Dimitri et al., 2018). This trend has only continued in recent years, with the vast majority of contemporary inpatient group research conducted outside the U.S., mostly in Germany (Weber & Strauss, 2015). Much of this research is on psychodynamic-oriented groups (Radcliffe & Diamond, 2007; Strauss & Schmidt, 2020), which is the dominant treatment model in Germany and was the primary model of inpatient group therapy in the U.S. in the 1970s/80s, whereas cognitive-behavioral therapy (CBT) has become the main treatment model in the U.S. in recent decades (Emond & Rasmussen, 2012). Consequently, past research in the U.S. and more recent research conducted in other countries has limited relevance to contemporary inpatient treatment in the U.S., where the average length of stay is magnitudes shorter than the group interventions utilized in these studies.

Length of stay is not the only factor rendering much of the research on inpatient group therapy irrelevant to contemporary inpatient treatment in the U.S. The vast majority of studies involved therapy groups that were homogeneous with respect to diagnosis and/or severity, with several studies conducted on specialized units (e.g., for substance use or eating disorders), and followed a closed-group structure (i.e., all members enter group on the same day and complete the series of group sessions together; Cook, Arechiga, Dobson, & Boyd, 2014; Emond & Rasmussen, 2012). In practice, inpatient groups tend to be highly heterogeneous both with respect to diagnosis and level of functioning. In the standard 24-bed psychiatric unit, there are simply not enough patients to split up into multiple specialized groups (Yalom, 1983). Additionally, ultra-short stays and quick patient turnover make it nearly impossible to have closed multi-session therapy groups with the same members (Emond & Rasmussen, 2012). Differences in the timing of admission and duration of stay mean that, on any given day, a different composition of patients will attend group, and individual patients will receive different exposures to group (different duration and/or modules). This is so much the case that Yalom suggests that inpatient group leaders “assume that your group will last for only a single session” (Yalom & Leszcz, 2020, p. 596), which is echoed in others’ calls for a stand-alone session format for inpatient groups (Cook, Arechiga, Dobson, & Boyd, 2014; Heriot-Maitland, Vidal, Ball, & Irons, 2014; Raune & Daddi, 2011; Snyder, Clark, & Jones, 2012). A survey of inpatient psychologists found that group heterogeneity and rapid turnover were among the main barriers to adapting evidence-based treatments for the inpatient setting (Snyder, Clark, & Jones, 2012).

The very small literature from the past 20 years that does exist serves to engender optimism for the benefits of group therapy adapted from outpatient EBTs for ultra-brief inpatient care. First, a few studies have found positive results for patient satisfaction with inpatient groups based on CBT, specifically in heterogeneous, rolling attendance groups (Boynton & Sanderson, 2022; Esposito-Smythers, McClung, & Fairlie, 2006; Heriot-Maitland, Vidal, Ball, & Irons, 2014; Nikolitch et al., 2016; Raune & Daddi, 2011). CBT is largely considered the most EBT model for briefer inpatient groups (David, Cristea, & Hofmann, 2018). A smaller body of research suggests that psychodynamic-oriented inpatient groups have also demonstrated positive effects in longer group formats (Radcliffe & Diamond, 2007; Strauss & Schmidt, 2020). A few studies have examined outcomes beyond feasibility and acceptability, observing reductions in symptoms (e.g., eating pathology, distress) and hospital re-admission (Gaudiano et al., 2020; Gaudiano et al., 2023; Veltro et al., 2006; Veltro et al., 2008; Wiseman, 2002). However, just three of these studies involved heterogeneous, rolling attendance groups, one of which studied an intervention involving both group and individual therapies (Gaudiano et al., 2023), and two reported on the same clinical trial conducted in Italy (Veltro et al., 2006; Veltro et al., 2008), where the average hospital stay is >2 weeks (Dimitri et al., 2018). Finally, there is some precedent for training inpatient staff, and specifically nondoctoral level staff, to deliver inpatient groups based on EBTs with adequate fidelity (Gaudiano et al., 2020; Gaudiano et al., 2023; Tyrberg, Carlbring, & Lundgren, 2017). This is clearly a very limited pool of research, such that it is easier to summarize what has been studied than to identify gaps that need to be filled. No study has involved an inpatient group model that addresses all of the important dimensions that distinguish contemporary inpatient care in the U.S., such as ultra-brief stays, quick patient turnover, group heterogeneity, and reliance on nonpsychologist group leaders.

## Conclusion

Inpatient hospitalization is one of the most critical periods for psychological intervention, and yet, research informing the main modality of psychological treatment in inpatient care, group therapy, is woefully lacking. Inpatient treatment in the U.S. has undergone such a substantial evolution over the past 50 years that research has not kept up with the pace of change, and group leaders are left with almost no relevant empirical guidelines for treatment. The primary purpose of this article is to serve as a call to action, eliciting increased efforts to study adaptations of psychological EBTs for the current ultra-brief inpatient treatment era in the U.S. Such interventions should account for the unique challenges of contemporary inpatient treatment, such as a focus on transdiagnostic models to meet heterogeneous patient needs and stand-alone session design to accommodate rolling attendance. Moreover, it is critical that research on inpatient groups employ study clinicians who reflect the staff that are most likely to be providing group therapy (e.g., social workers and psychiatric nurses). This will likely require initial efforts to develop and study training programs designed to provide multidisciplinary staff with the necessary background in EBTs. Initiatives at lower levels of care, such as the Project Air Strategy for Personality Disorders in Australia (Grenyer, 2014), provide a model for developing “easy to learn” interventions that multidisciplinary healthcare workers can deliver. Although patients’ time on the unit is short, much of that time is spent in group therapy, which for some individuals will be the only exposure to psychotherapy they ever have. We should make sure that time counts!

## References

[r1] Adepoju, O. E., Kim, L. H., & Starks, S. M. (2022). Hospital length of stay in patients with and without serious and persistent mental illness: Evidence of racial and ethnic differences. Healthcare, 10(6), 1128. 35742179 10.3390/healthcare10061128 PMC9223052

[r2] Badano, V. (2024). The Basaglia Law. Returning dignity to psychiatric patients: The historical, political and social factors that led to the closure of psychiatric hospitals in Italy in 1978. History of Psychiatry, 35(2), 226–233. 38334117 10.1177/0957154X231224650 PMC11092286

[r3] Berry, K., Raphael, J., Haddock, G., Bucci, S., Price, O., Lovell, K., Drake, R.J., Clayton, J., Penn, G. & Edge, D. (2022). Exploring how to improve access to psychological therapies on acute mental health wards from the perspectives of patients, families and mental health staff: Qualitative study. BJPsych Open, 8(4), e112. 35698827 10.1192/bjo.2022.513 PMC9230441

[r4] Boynton, V., & Sanderson, C. (2022). Open group cognitive behaviour therapy on acute in-patient units. Behavioural and Cognitive Psychotherapy, 50(1), 106–110. 33468278 10.1017/S1352465821000011

[r5] Burlingame, G. M., Fuhriman, A., & Mosier, J. (2003). The differential effectiveness of group psychotherapy: A meta-analytic perspective. Group Dynamics: Theory, Research, and Practice, 7(1), 3.

[r6] Cook, W. G., Arechiga, A., Dobson, L. A. V., & Boyd, K. (2014). Brief heterogeneous inpatient psychotherapy groups: A process-oriented psychoeducational (POP) model. International Journal of Group Psychotherapy, 64(2), 180–206. 24611701 10.1521/ijgp.2014.64.2.180

[r7] Cromwell, J., Maier, J., Gage, B., Drozd, E., Osber, D., Richter, E., … Goldman, H. (2004). Characteristics of high staff intensive medicare psychiatric inpatients. Health Care Financing Review, 26(1), 103. 15776703 PMC4194884

[r8] Dandan, N., Mansour, F., & Diab, T. (2024). The role of psychology in a multi-disciplinary psychiatric inpatient setting: Perspectives from the multidisciplinary team. Middle East Current Psychiatry, 31(1), 26.

[r9] David, D., Cristea, I., & Hofmann, S. G. (2018). Why cognitive behavioral therapy is the current gold standard of psychotherapy. Frontiers in Psychiatry, 9, 4. 29434552 10.3389/fpsyt.2018.00004 PMC5797481

[r10] Diefenbach, G. J., Lord, K. A., Stubbing, J., Rudd, M. D., Levy, H. C., Worden, B., … Everhardt, K. (2024). Brief cognitive behavioral therapy for suicidal inpatients: A randomized clinical trial. JAMA Psychiatry, 81(12), 1177–1186. 39259550 10.1001/jamapsychiatry.2024.2349 PMC11391362

[r11] Dimitri, G., Giacco, D., Bauer, M., Bird, V. J., Greenberg, L., Lasalvia, A., Lorant, V., Moskalewicz, J., Nicaise, P., Pfennig, A., Ruggeri, M., Welbel, M. & Priebe, S. (2018). Predictors of length of stay in psychiatric inpatient units: Does their effect vary across countries? European Psychiatry, 48(1), 6–12. 29331601 10.1016/j.eurpsy.2017.11.001

[r12] Emond, S., & Rasmussen, B. (2012). The status of psychiatric inpatient group therapy: Past, present, and future. Social Work with Groups, 35(1), 68–91.

[r13] Esposito-Smythers, C., McClung, T. J., & Fairlie, A. M. (2006). Adolescent perceptions of a suicide prevention group on an inpatient unit. Archives of Suicide Research, 10(3), 265–275. 16717043 10.1080/13811110600582554

[r14] Evlat, G., Wood, L., & Glover, N. (2021). A systematic review of the implementation of psychological therapies in acute mental health inpatient settings. Clinical Psychology & Psychotherapy, 28(6), 1574–1586. 33870590 10.1002/cpp.2600

[r15] Fakhoury, W., & Priebe, S. (2002). The process of deinstitutionalization: An international overview. Current Opinion in Psychiatry, 15(2), 187–192.

[r16] Frank, H. E., Becker-Haimes, E. M., & Kendall, P. C. (2020). Therapist training in evidence-based interventions for mental health: A systematic review of training approaches and outcomes. Clinical Psychology: Science and Practice, 27(3), e12330. 34092941 10.1111/cpsp.12330 PMC8174802

[r17] Gaudiano, B. A., Ellenberg, S., Johnson, J. E., Mueser, K. T., & Miller, I. W. (2023). Effectiveness of acceptance and commitment therapy for inpatients with psychosis: Implementation feasibility and acceptability from a pilot randomized controlled trial. Schizophrenia Research, 261, 72–79. 37716204 10.1016/j.schres.2023.09.017 PMC10841307

[r18] Gaudiano, B. A., Ellenberg, S., Ostrove, B., Johnson, J., Mueser, K. T., Furman, M., & Miller, I. W. (2020). Feasibility and preliminary effects of implementing acceptance and commitment therapy for inpatients with psychotic-spectrum disorders in a clinical psychiatric intensive care setting. Journal of Cognitive Psychotherapy, 34(1), 80. 32701478 10.1891/0889-8391.34.1.80 PMC8965771

[r19] Geller, J. L., Fisher, W. H., McDermeit, M., & Brown, J. M. (1998). The effects of public managed care on patterns of intensive use of inpatient psychiatric services. Psychiatric Services, 49(3), 327–332. 9525791 10.1176/ps.49.3.327

[r20] Glick, I. D., Sharfstein, S. S., & Schwartz, H. I. (2011). Inpatient psychiatric care in the 21st century: The need for reform. *Psychiatric Services*. 10.1176/ps.62.2.pss6202_0206 21285100

[r21] Grenyer, B. F. (2014). An integrative relational step-down model of care: The project air strategy for personality disorders. The ACPARIAN, 9, 8–13.

[r22] Heriot-Maitland, C., Vidal, J. B., Ball, S., & Irons, C. (2014). A compassionate-focused therapy group approach for acute inpatients: Feasibility, initial pilot outcome data, and recommendations. British Journal of Clinical Psychology, 53(1), 78–94. 24588763 10.1111/bjc.12040

[r23] Kösters, M., Burlingame, G. M., Nachtigall, C., & Strauss, B. (2006). A meta-analytic review of the effectiveness of inpatient group psychotherapy. Group Dynamics: Theory, Research, and Practice, 10(2), 146.

[r24] Lave, J. R. (2003). Developing a medicare prospective payment system for inpatient psychiatric care. Health Affairs, 22(5), 97–109. 10.1377/hlthaff.22.5.97 14515885

[r25] Lutterman, T. (2022). Trends in psychiatric inpatient capacity, united states and each state, 1970 to 2018. In Technical assistance collaborative paper no 2 Alexandria, VA: National Association of State Mental Health Program Directors.

[r26] Medicare, Medicaid, and SCHIP Balanced Budget Refinement Act of 1999 (1999). Pub. L. No. 106–113 § 124.

[r27] Nikolitch, K., Laliberté, V., Yu, C., Strychowsky, N., Segal, M., Looper, K. J., & Rej, S. (2016). Tolerability and suitability of brief group mindfulness-oriented interventions in psychiatric inpatients: A pilot study. International Journal of Psychiatry in Clinical Practice, 20(3), 170–174. 27334931 10.1080/13651501.2016.1197276

[r28] Olfson, M., Marcus, S. C., & Doshi, J. A. (2010). Continuity of care after inpatient discharge of patients with schizophrenia in the Medicaid program: A retrospective longitudinal cohort analysis. The Journal of Clinical Psychiatry, 71(7), 3717. 10.4088/JCP.10m05969yel 20441730

[r29] Paterson, C., Karatzias, T., Dickson, A., Harper, S., Dougall, N., & Hutton, P. (2018). Psychological therapy for inpatients receiving acute mental health care: A systematic review and meta-analysis of controlled trials. British Journal of Clinical Psychology, 57(4), 453–472. 29660770 10.1111/bjc.12182

[r30] Pudalov, L. R., Swogger, M. T., & Wittink, M. (2018). Towards integrated medical and mental healthcare in the inpatient setting: What is the role of psychology? International Review of Psychiatry, 30(6), 210–223. 30821187 10.1080/09540261.2018.1552125

[r31] Radcliffe, J., & Diamond, D. (2007). Psychodynamically-informed discussion groups on acute inpatient wards. Groupwork, 17(1), 34–44.

[r32] Raune, D., & Daddi, I. (2011). Pilot study of group cognitive behaviour therapy for heterogeneous acute psychiatric inpatients: Treatment in a sole-standalone session allowing patients to choose the therapeutic target. Behavioural and Cognitive Psychotherapy, 39(3), 359–365. 21320360 10.1017/S1352465810000834

[r33] Salinsky, E., & Loftis, C. (2007). Shrinking inpatient psychiatric capacity: Cause for celebration or concern? National Health Policy Forum. Paper 183. https://hsrc.himmelfarb.gwu.edu/sphhs_centers_nhpf/183 17679175

[r34] Scaturo, D. J. (2004). Fundamental clinical dilemmas in contemporary group psychotherapy. Group Analysis, 37(2), 201–217.

[r35] Shah, B., Leontieva, L., & Megna, J. L. (2020). Shifting trends in admission patterns of an acute inpatient psychiatric unit in the state of New York. Cureus, 12(7), e9285. 32832283 10.7759/cureus.9285 PMC7437124

[r36] Sharfstein, S. S. (2009). Goals of inpatient treatment for psychiatric disorders. Annual Review of Medicine, 60(1), 393–403. 10.1146/annurev.med.60.042607.080257 18729730

[r37] Smith, T. E., Abraham, M., Bolotnikova, N. V., Donahue, S. A., Essock, S. M., Olfson, M., … Radigan, M. (2017). Psychiatric inpatient discharge planning practices and attendance at aftercare appointments. Psychiatric Services, 68(1), 92–95. 27582241 10.1176/appi.ps.201500552

[r38] Snyder, J. A., Clark, L. M., & Jones, N. T. (2012). Provision and adaptation of group treatment in state psychiatric hospitals. Professional Psychology: Research and Practice, 43(4), 395.

[r39] Stein, B. D., Kogan, J. N., Sorbero, M. J., Thompson, W., & Hutchinson, S. L. (2007). Predictors of timely follow-up care among Medicaid-enrolled adults after psychiatric hospitalization. Psychiatric Services, 58(12), 1563–1569. 18048557 10.1176/ps.2007.58.12.1563

[r40] Strauss, B., & Schmidt, S. (2020). Differential treatment outcome of inpatient psychodynamic group work. In Research on psychoanalytic psychotherapy with adults (pp. 15–34): Routledge.

[r41] Szabo, C. P., & Kaliski, S. Z. (2017). Mental health and the law: A South African perspective. BJPsych International, 14(3), 69–71. 29093950 10.1192/s2056474000001951 PMC5618904

[r42] Taborda, J. G. (2013). Mental health law in Brazil. InternatIonal Psychiatry, 10(1), 13–15. 31507716 PMC6735106

[r43] Tulloch, A. D., Fearon, P., & David, A. S. (2011). Length of stay of general psychiatric inpatients in the United States: Systematic review. Administration and Policy in Mental Health and Mental Health Services Research, 38, 155–168. 20924662 10.1007/s10488-010-0310-3

[r44] Turner, T. (2004). The history of deinstitutionalization and reinstitutionalization. Psychiatry, 3(9), 1–4.

[r45] Tyrberg, M. J., Carlbring, P., & Lundgren, T. (2017). Implementation of acceptance and commitment therapy training in a psychiatric ward: Feasibility, lessons learned and potential effectiveness. Journal of Psychiatric Intensive Care, 13(2), 73–82.

[r46] Tyrberg, M. J., & Klintwall, L. (2022). Transdiagnostic ultra-brief behaviour therapy for psychiatric inpatients: A multiple-baseline single-case design. Journal of Psychiatric Intensive Care, 18(2), 83–94.

[r47] Veltro, F., Falloon, I., Vendittelli, N., Oricchio, I., Scinto, A., Gigantesco, A., & Morosini, P. (2006). Effectiveness of cognitive-behavioural group therapy for inpatients. Clinical Practice and Epidemiology in Mental Health, 2, 1–6. 16859548 10.1186/1745-0179-2-16 PMC1552055

[r48] Veltro, F., Vendittelli, N., Oricchio, I., Addona, F., Avino, C., Figliolia, G., & Morosini, P. (2008). Effectiveness and efficiency of cognitive-behavioral group therapy for inpatients: 4-year follow-up study. Journal of Psychiatric Practice®, 14(5), 281–288. 18832959 10.1097/01.pra.0000336755.57684.45

[r49] Ward-Ciesielski, E. F., & Rizvi, S. L. (2021). The potential iatrogenic effects of psychiatric hospitalization for suicidal behavior: A critical review and recommendations for research. Clinical Psychology: Science and Practice, 28(1), 60.

[r50] Weber, R., & Strauss, B. (2015). Group psychotherapy in Germany. International Journal of Group Psychotherapy, 65(4), 513–525. 26401794 10.1521/ijgp.2015.65.4.513

[r51] Wiseman, C. V. (2002). Short-term group CBT versus psycho-education on an inpatient eating disorder unit. Eating Disorders, 10(4), 313–320. 16864274 10.1080/10640260214504

[r52] Yalom, I. D. (1983). Inpatient group psychotherapy: Basic Books.

[r53] Yalom, I. D., & Leszcz, M. (2020). The theory and practice of group psychotherapy. Basic Books.

